# The Food and the Teeth

**Published:** 1865-10

**Authors:** James Paul

**Affiliations:** Trenton, N.J.


### THE FOOD AND THE TEETH.—OBSERVATIONS ON THE INORGANIC CONSTITUENTE OF THE FOOD OF CHILDREN, AS CONNECTED WITH THE DECAY OF THE TEETH, AND THE PHYSICAL CONSTITUTION OF WOMEN IN AMERICA.

BY the late JAMES PAUL, M.D., of Trenton, N.J.

[The following very valuable and instructive paper was read before the District Medical Society, for the County of Mercer, N.J., some years ago, by the late JAMES PAUL, M.D., of Trenton. There is so much in it worthy the consideration of the profession and the public, that we deem it of sufficient importance to reproduce it.—*Ed. Med. and Sur. Rep.*]

The subject to which I have the pleasure of directing your attention is, not only in a physiological point of view, one of interest, but in its application to the preservation of health—the tendency to improve the general condition and physical constitution of the human family inhabiting this great continent—a continent abounding, as it does, in all the productions which a bountiful Creator, in his beneficience, bestows on man—cannot be otherwise than of great and paramount importance.

At a period somewhat now remote, the celebrated naturalist,

Buffon, alluding to the animals of this continent, advanced the following opinions:—

1st. That the animals common both to the Old and New Worlds are smaller in the latter.

2d. That those belonging to the New are on a smaller scale.

3d. That those which have been domesticated in both, have degenerated in America.

4th. That, on the whole, it exhibits fewer species.

These opinions, Mr. Jefferson, in his "Notes on Virginia," undertook, and it is generally considered successfully, to controvert; yet, however repugnant to the general idea the opinion as to the tendency of those animals which have been domesticated in America from other countries to degenerate, it is an undeniable and much to be regretted fact, that the human family, and more particularly the female portion of that family, have declined in the vigor and strength of their physical constitution.

I wish not to be misunderstood: I say it is a melancholy fact, too well known to the observant physiologist, that increase of strength, and development of frame, have not been attained by the intermarrying of members of the human family of different nations on this continent; but the reverse is too observable; the physical frame of the female sex has degenerated—calling loudly for the aid of science to arrest an evil of so much magnitude.

Let us for a moment contemplate the female form, as seen on this broad continent. In no country in the world are children more fair and beautiful; and as the young girl grows up to womanhood, we see in her a full realization of that being forming in the hands of Divinity, portrayed by the poet, as seen by Adam in his dream:—

> "Under his forming hands, a creature grew,
> Manlike, but different sex; so lovely fair,
> That what seemed fair in all the world, seemed now
> Mean, or in her summed up, in her contained,
> And in her looks;"—

We see this young and lovely being—the forehead well developed—the countenance, rather elongated, relieved of the harsher outline of some of the European nations—with fragile form, and small, yet well-developed bust, fliting for a few short years among us, and then—yes, then there comes a change. Ere five and twenty summers pass, this flower begins to fade—the rounded form shrinks—the bloom of health decays; and if she escapes the fell destroying angel's death-like grasp, a wreck of former self remains.

Why should this be so? The robust of other countries come to this continent—they live in comfort—their food is excellent in quality—their progeny is like themselves—but even now, in the very first generation, does the degenerating process make itself manifest—the teeth begin to decay; and girls, while yet children, have to visit the dentist to have them cleansed, scraped, and plugged.

Now this brings us at once to the head and front of our subject; and if we can point out the first cause of this decay of what should be as strong as adamant, it may be the means in helping us in our investigation. That there is something radically wrong in our system of rearing the young, to which this misfortune is in a great measure owing, I am free to confess, is my firm opinion. I would indeed it were in my power, in pointing out the evil, to be as successful in detailing the cause, that we may apply the remedy. Still, although perhaps unable to accomplish all I wish, my observations may not be without their weight, and induce others, more observant, more scientific, and more competent to the task, to follow up an investigation so fraught with advantage to our fellow-beings.

It is certainly to be deplored that the females of this continent, descendents of European parents, should be so much afflicted with caries of the teeth—the decay of parts formed of substances which enter into the composition of some of our hardest minerals—marble, bone-earth and fluorspar; and this decay unfortunately occurs in early life—in girls yet at school; and many a young woman, ere she has attained a marriageable age, has had to replace the natural with the unnatural, though more enduring enamel of the artist's formation. This ought not to be: God made all mankind alike; in no portion of the earth are nations found who lose their hands, or feet, or tongue, er eyes; and there can be no cause why the inhabitants of this land should loose their teeth. It is not so in the olden countries from whence the progenitors of the present race have come; nor is it so in the West India Islands, which may almost be considered as part of this great continent. So excellent is the structure of the teeth of savage nations, that some tribes in Africa, I think the Mocoes and Mundingoes, file all the front teeth, so that they shall be separated and form sharp points, the better to tear the uncooked animal food.

One cause of this affliction is, in the mind of many, attributed to the great and sudden changes of temperature experienced on this continent—the thermometer rising and falling 20, 30, and even 40 degrees in twelve hours. But if attributable to these sudden changes, we know that sudden expansion by means of heat, or sudden contraction by means of cold, causing the particles of which bodies are composed to tear themselves asunder; consequently to crack, break, and fall in pieces. But this is not the case with the teeth of our females; a caries or decay commences most generally in the side of the tooth, extending to the enamel, which is sometimes involved in the destruction; at other times, it is left a crust or shell to snap and break off in small pieces, when unable to resist the pressure of whatever may be placed against it; besides, the teeth are for the most part sheltered from these sudden changes, and kept at a temperature nearly amounting to blood-heat at all seasons. I do not think we can place the general destruction of the teeth, and consequent affliction of the females of America to this cause. I fear we must rather look for it to constitutional weakness, and this constitutional weakness to a deficiency of the inorganic or earthy constituents being taken into the system, more particularly at an early period of life.

If I am correct in this opinion, and reason, philosophy, and a thorough examination of physiological facts in both the animal and vegetable economy, tend far to bear out these views, then if we would try and correct this lamentable state of things, let us commence at the very beginning, and make ourselves acquainted by examining the structure and composition of the teeth, and then we shall be more able to understand what is required to aid nature in their formation and consequent preservation.

First, then, let us make ourselves acquainted with the structure and composition of the teeth. The teeth are nearly allied to bone in structure; both having earthy deposits, intermixed with fibres and cells of gelatine, which, by consolidation, gives form and strength—in the case of bone, to bear the weight of the various parts, and afford protection to the different organs of the body; and in the case of teeth, to cut and grind the food required for the formation, support, and reparation of its various parts.

Now, teeth are composed of three different substances, and these three are disposed according to the purposes required of them; they are, *cementum* or *crusta petrosa*, *dentine* (known as ivory in the tusk of the elephant), and *enamel*. The *cementum* or *crusta petrosa*, corresponds in all especial particulars with bone; possessing its characteristic *lucunæ* or small cavities, and being traversed by vascular and medulary canals, whenever it occurs of sufficient thickness; it is the first covering of the young teeth, and may be said to invest the fang of the tooth which enters the aveolar process of the jaw. The *dentine*, or ivory, consists of a firmer substance, in which inorganic or mineral matter predominates, though to a less degree than *enamel*. It is traversed by a vast number of very fine cylindrical, branching wavy tubuli, which commence at the pulpy cavity, and radiate toward the surface. The diameter of these tubuli, at their largest part, average about one 10,000th of an inch: their smallest are immeasurably fine; so much so that they cannot possibly receive blood, but it is surmised that, like the canaliculi of bone, they imbibe fluid from the vascular lining of the pulp-cavity, which aids in the nutrition of the tooth. The *enamel* is composed of solid prisms of fibres, about the one 5,600th of an inch in diameter, arranged side by side, and closely adherent to each other; their length corresponds with the thickness of the layer which they form; and the two surfaces of this layer present the ends of the prism, which are usually more or less hexagonal. In the perfect state, the enamel contains but an extremely minute quantity of animal matter. In the centre of the tooth is the soft pulpy cavity, which affords a bed for the bloodvessels and nerves which supply it with life and sensibility.

I shall not enter more minutely into the structure of the teeth, but may briefly state, that like all other structures of the animal body, the component parts are derived and deposited from the blood, by that mysterious and incomprehensible power that selects and deposits the necessary constituents in the formation of the several portions, according to the use required.

Now, in the composition of the teeth, we have first the division into organic and inorganic or earthy matter; and we find that the several substances which enter into the structure of the teeth, differ chiefly as to the earthy matter contained in each.

Chemical analysis of the incisors, or front teeth of man, show that they contain in one hundred parts of each, as follows:—

| | Cementum. | Dentine. | Enamel. |
|---|---|---|---|
| Organic Matter | 29.27 | 28.70 | 3.59 |
| Earthy Matter | 70.73 | 71.30 | 96.41 |
| | 100. | 100. | 100. |

These proportions will occasionally differ; in some individuals the organic constituents having less than here stated, amounting in the dentine only to 21. The analysis of bone, however, gives a much larger proportion, viz.:—

| | |
|---|---|
| Organic matter | 32.56 |
| Earthy matter | 67.44 |
| | 100. |

Let us now take a more complete analysis, showing what earthy constituents enter into their composition. Analysis of the molar or grinding teeth of man, and of the bones of the arm and leg of a man of forty, show the following proportions:

| | Dentine. | Enamel. | Bone. |
|---|---|---|---|
| Inorganic Matter:— | | | |
| Phosphate of Lime with traces of Fluate of Lime | 66.72 | 89.82 | 54.61 |
| Carbonate of Lime | 3.36 | 4.37 | 9.41 |
| Phosphate of Magnesia | 1.08 | 1.34 | 1.07 |
| Salts, etc. | .83 | .88 | 2.35 |
| Organic Matter | 28.01 | 3.59 | 32.56 |
| | 100. | 100. | 100. |

Thus we see the very great proportion of certain earths that enter into the structure of the teeth and bone of man, the chief substance being the phosphate of lime, familiarly known as bone earth. We find, too, that whereas in ordinary bone the phosphate of lime constitutes only 54 parts in 100, in the enamel of the teeth it is nearly 90 parts in 100—while the carbonate of lime in bone amounts to 9.41, in the enamel of teeth it is only 4.37; the enamel being literally almost a mineral in substance, having only 3.59 parts of animal matter in 100.

Thus the teeth to be strong and durable, require a large quantity of earthy ingredients, particularly *lime*, to enter into their composition. Let us inquire whence it is derived; and for this we must examine the blood.

To allow of such deposits from the blood, it is first necessary that they should be held in solution in that fluid. You are no doubt aware that the blood circulates to every portion of the body by the action of the heart, which forces a certain quantity, say 2 oz. at every contraction, into the aorta or great canal leading from the left ventricle—that the aorta divides and subdivides into innumerable branches, which are made to ramify to every part of the body, until the extreme branches end in capillary tubes or vessels, the calibre of which is so small as not to allow the red globules or corpuscles of the blood to enter them, but which allows the serous portion to traverse every part of the organized structure, holding in solution all those constituents necessary and requisite for the formation and reparation of its several parts.

In the serous portion of the blood, then, we find contained the constituents required for the composition of bone and teeth —analysis of 1000 parts of healthy human blood giving, according to M. Lecanu, the following proportions:—

| | | |
|---|---|---|
| Water | 780.15 | 785.58 |
| Fibrine | 2.10 | 3.57 |
| Albumen | 65.09 | 69.41 |
| Coloring matter | 133.00 | 119.63 |
| Crystalizable fat | 2.43 | 4.30 |
| Fluid fat | 1.31 | 2.27 |
| Extractive matter, uncertain | 1.79 | 1.92 |
| Albumen in combination with Soda | 1.26 | 2.01 |
| Chlorides of Sodium and Potassium; Carbonates, Phosphates, and Sulphates of Potash and Soda | 8.37 | 7.30 |
| Carbonates of Lime and Magnesia; Phosphates of Lime, Magnesia, and Iron; Peroxide of Iron | 2.10 | 1.42 |
| Loss | 2.40 | 2.50 |
| | 1000. | 1000. |

We see by this table, if we subtract or take away the proportion of water amounting to 780 parts, and the coloring matter amounting to 133, we shall leave scarcely 90 parts of organic and earthy material, the salts and earths forming upwards of a 10th—the salts being in proportion to the earths as 4 to 1. Having then shown the constituent portions of the bones and teeth to be in the blood, the next consideration is, whence are they derived?

Before entering on this subject further, let us for a moment take a broader and more comprehensive view of what must be most interesting to mothers, and of great consequence to the well-being of the infant generation, in a short time, in a very few years, to become in their turn the mothers and fathers of another generation.

The question then presents itself, what is the nourishment or food best adapted and necessary to the wants of an infant, that the foundation may be laid for a strong frame and vigorous constitution? For here, we must recollect, is the starting point in by far the majority of instances. We know that in some cases disease is hereditary—that the offspring unfortunately inherits from the parents constitutional defects; but we also know that more misery, suffering, and constitutional derangement, are entailed on children by want of care, and improper food in the first years of life, by which their hopes of health are blasted, and they are doomed to struggle through a weary life, to be hurried at last into a premature grave.

Now, that the frame—that is, the bones, muscles, and other portions of the infant—may be fully developed, it is necessary that it should be supplied with nourishment, containing all the constituents required for this important undertaking. And this nourishment, by the all-wise ordering of Providence, is contained in the milk secreted from the mother's bosom.

The infant is entirely dependent on the nourishment derived from its mother, and nature has wisely ordained that the secretion from the mother is its very best food; for we find in the composition of milk—that is, healthy milk, derived from healthy blood—all those ingredients we have hitherto traced as requisite in the formation of the bones and teeth, and not only these, but every constituent required for the life and growth of the individual;—milk containing the albuminous, saccharine, oleaginous, saline, and earthy componds requisite and necessary for the health, strength, and development of the infant child.

How thankful ought we to be to the all-wise and bountiful Giver of all good, for this beneficient, this wonderful provision in nature, by which there shall be secreted from the mother, a fluid so important, having properties blended in intimate connection, to afford the requisite substances for the support, growth, and development of her offspring.

An analysis of cow's milk gives the following proportions of the various constituents; that of human milk is not so elaborate, but contains the average of observations taken at fourteen different times from the same individual, by Simon.

Cow's milk by M. Haidlen.

| | |
|---|---|
| Water | 873.00 |
| Butter | 30.00 |
| Caseine | 48.20 |
| Milk Sugar | 43.90 |
| Phosphate of Lime | 2.31 |
| Phosphate of Magnesia | .42 |
| Phosphate of Iron | .07 |
| Chloride of Potassium | 1.44 |
| Chloride of Sodium | .24 |
| Soda in connection with Caesine | .42 |
| | 1000. |

Woman's milk by Simon.

| | |
|---|---|
| Water | 883.6 |
| Butter | 25.3 |
| Caesine | 34.3 |
| Milk, Sugar, and Extractive Matter | 48.2 |
| Fixed Salts | 2.3 |
| | 1000. |

| | Maxium of 14 observations. | Minium of 14 observations. |
|---|---|---|
| Butter | 54.0 | 8.0 |
| Caseine | 45.2 | 10.6 |
| Sugar and Extractive Matter | 62.4 | 39.2 |
| Salts | 2.7 | 1.6 |

Now, although these amounts will no doubt vary, under every variety of circumstances, according to the *health*, *exercise*, *passions* and *food* of the mother, yet they show what I particularly wish to impress on your minds, that healthy milk contains all the requisites for the nourishment of the infant—but then it must be *healthy* milk, secreted from healthy blood, and that blood must derive these ingredients from the *food* consumed, otherwise they will be taken up from the structures of the body, and hence the havoc made in nursing females when a due allowance of proper aliment is withheld, and the shrunken body of the famished mother is drained to the last drop, to supply the cravings of the death-like and impoverished offspring.

I have said that the composition of milk in quality and quantity, will vary and depend on circumstances. Now, the mental state exerts a surprising influence on this secretion, and much more than is usually supposed. It may not be irrelevant to mention a few of the cases recorded in our journals,* of the influence of strong mental excitement on this secretion.

"A carpenter fell into a quarrel with a soldier billeted in his house, and was set upon by the latter with his drawn sword. The wife of the carpenter, at first, trembled from fear and terror, and then suddenly threw herself furiously between the combatants, wrested the sword from the soldier's hand, broke it in pieces, and threw it away. During the tumult, some neighbors came in and separated the men. While in this state of strong excitement, the mother took up her child from the cradle, where it lay playing, and in the most perfect health, never having had a moment's illness; she gave it the breast, and, in

* From Carpenter's Physiology.

so doing, sealed its fate. In a few minutes the infant left off sucking, became restless, panted, and sank dead upon its mother's bosom. The physician, who was instantly called in, found the child lying in the cradle, as if asleep, and with its features undisturbed; but all his resources were fruitless. It was irrevocably gone."

"A lady having several children, of which none had manifested any particular tendency to cerebral disease, and of which the youngest was a healthy infant a few months old, heard of the death of the infant child of a friend residing at a distance, with whom she had been on terms of close intimacy, and whose family had increased cotemporaneously with her own. The circumstance naturally made a strong impression on her mind, and she dwelt upon it the more, perhaps, as she happened at that period to be separated from the rest of her family, and to be much alone with her babe. One morning shortly after having nursed it, she laid it in its cradle, asleep and apparently in perfect health; her attention was shortly attracted to it by a noise, and on going to the cradle, she found her infant in a convulsion, which lasted for a few minutes, and left it dead."

"A mother had lost several children in early infancy from a convulsive disorder. One infant, however, survived the usual fatal period; but whilst nursing him one morning, she had been strongly dwelling on the fear of losing him also, although he appeared a very healthy child. In a few minutes after the infant had been transferred into the arms of the nurse, and while she was urging her mistress to take a more cheerful view, directing her attention to his thriving appearance, he was seized with a convulsion-fit, and died almost instantly."

These are interesting cases, and tend to show the great influence the mental affections exert on the secretion of milk, in rendering it deleterious in quality, and unwholesome to the infant.

Returning then to our subject, you will observe by the analysis, that cow's milk differs from that of woman in the proportions of some of the constituents, that it abounds more in butter, but particularly in caseine or cheese; and on the other hand, that human milk abounds more in the saccharine principle, or sugar of milk. Now, this points out a circumstance from which great benefit may be derived. It is of very frequent occurrence that infants are deprived of the natural nourishment of the mother, and diverse opinions are given relative to the food of infants by persons who really know very little about the matter; one recommends a milk diet, another that the infant must be fed on starch and sugar.

Now, to enable the infant to receive a nourishment in every respect similar to the mother, the knowledge of the various proportions which we obtain by chemical analysis, enables us to rectify and produce milk very analogous to human milk from that of the cow, by diluting it with water, in the proportion of about half as much again; that is, to a pint of milk should be added half a pint of water that has been boiled, which will reduce the cheese principle to the proper proportion; add a small portion of cream to restore the proportion of butter, and then add sugar until the whole is distinctly sweetened, and we have a compound, in every respect, similar to the milk from the human breast.

To understand the subject of nutrition, allow me to explain to you, that food should, or must embody two great principles, one to nourish, the other to give heat to the body. And food, when consumed, is applied to one or the other of these purposes. Now, in the process of digestion, the constituents of the food are separated, and arranged in three classes.

1st. All that portion derived from animal food, eggs, the curd of milk, the gluten or adhesive portion of wheat and other grain, and whatever in animal or vegetable food can be rendered into *albumen*—of which the best example that can be offered in illustration is the *white of egg*, which is, in reality, nearly pure albumen—and the principle is, therefore, called *albuminous*.

2d. All that portion of the food derived from vegetables, starch, sugar, etc., that can be converted into *sugar* in the process of digestion. This principle is, therefore, called *saccharine*.

3d. All the fat, butter, oil, etc., which, when deprived of the other substances is left in the state of *oil*, and, therefore, called *oleaginous*.

Now, of these three, the *albuminous* is the *nutrient*, and the *saccharine* and *oleaginous* the *calorifacient*, or heat giving; and chemical analyses show that they vary in composition.

| | ALBUMEN. | | OLEAGINOUS. |
|---|---|---|---|
| | Eggs. | Wheat. | Mutton Fat. |
| Carbon | 55,000 | 55.01 | 78,996 |
| Hydrogen | 7,073 | 7.23 | 11,700 |
| Nitrogen | 15,920 | 15.92 | |
| Oxygen } | | | 9,304 |
| Sulphur } | 22,007 | 21.84 | |
| Phosphorus } | | | |

| SACCHARINE. | Starch, Arrow Root. | Sugar from Starch. | Sugar of Milk. | Cane Sugar. |
|---|---|---|---|---|
| Carbon | 44.10 | 37.29 | 40.00 | 42.301 |
| Hydrogen | 6.18 | 6.84 | 6.61 | 6.384 |
| Oxygen | 49.42 | 55.87 | 52.93 | 51.315 |

[*Continued in the next No.*]

